# Comparison between Dexmedetomidine and Midazolam for Sedation in Patients with Intubation after Oral and Maxillofacial Surgery

**DOI:** 10.1155/2020/7082597

**Published:** 2020-04-08

**Authors:** Li Wang, Tiejun Zhang, Lili Huang, Wei Peng

**Affiliations:** ^1^The State Key Laboratory Breeding Base of Basic Science of Stomatology (Hubei-MOST) and Key Laboratory of Oral Biomedicine Ministry of Education, School and Hospital of Stomatology, Wuhan University, Wuhan, Hubei 430079, China; ^2^Department of Anesthesiology, School and Hospital of Stomatology, Wuhan University, Wuhan, Hubei 430079, China

## Abstract

The aim of the investigation is to clarify the beneficial sedative effects for patients with postoperative intubation in the intensive care unit (ICU) after oral and maxillofacial surgery. Forty patients with postoperative intubation were divided into two groups in method of random number table: midazolam group and dexmedetomidine group. The Ramsay score, the behavioral pain scale (BPS) score, SpO_2_, HR, MAP, and RR were recorded before sedation (T0), 30 minutes (T1), 1 hour (T2), 2 hours (T3), 6 hours (T4), and 12 hours (T5) after dexmedetomidine or midazolam initiation in intensive care unit, and 10 minutes after extubation (T6). The rate of incidences of side effects was calculated. Sedation with midazolam was as good as standard sedation with dexmedetomidine in maintaining target sedation level. The BPS score in the midazolam group was higher than that in the dexmedetomidine group. The time of tracheal catheter extraction in the dexmedetomidine group was shorter than that in the midazolam group (*p* ≤ 0.001). The incidence of bradycardia in the dexmedetomidine group was higher than that in the midazolam group (*p* = 0.028). There was no statistically significant difference in the incidence of hypotension between the two groups (*p* = 0.732). The incidence of respiratory depression of group midazolam was higher than that of group dexmedetomidine (*p* = 0.018). The incidence of delirium in the dexmedetomidine group was significantly lower than that in the midazolam group, and the difference was statistically significant (*p* = 0.003). Dexmedetomidine and midazolam can meet the needs for sedation in ICU patients. And dexmedetomidine can improve patients' ability to communicate pain compared with midazolam.

## 1. Introduction

Because of being adjacent to the airway, oral and maxillofacial surgery is often accompanied with changes in oral anatomy and it is often complicated by edema, strictures, bleeding, increased hypopharyngeal secretions, and decreased pharyngeal airway-protective reflexes [1]. It is more common and serious to have an emergency airway than other surgeries, which may be life-threatening with early removal of the tracheal tube [2]. In order to ensure airway patency, the postoperative placement of tracheal tube is an ideal choice. But the patients find it often difficult to tolerate the tracheal tube, since it can cause cough, restlessness, and increased blood pressure and heart rate with other adverse reactions, which would not only cause adverse effects on the physiological function but also significantly delay the patient's rehabilitation process, and produce more serious surgical complications. Therefore, it is essential to use rational drugs to block these stress response and physiological changes, to improve the comfort of postoperative retention of tracheal catheter and prognosis, to reduce the incidence of postoperative complications, and to take effective sedative analgesic treatment.

Midazolam is commonly used in the ICU for sedation of the ventilated postsurgical patient [3]. Dexmedetomidine is a highly specific alpha-2-adrenergic receptor agonist that possesses sedative, anxiolytic, and analgesic effects. The comfort of patients can be effectively enhanced through using the analgesia-based sedation strategy [4, 5]. Hydromorphone can effectively control the pain in ICU, as it is a kind of *μ*-opioid receptor agonist [6]. In this study, we retrospectively compare the effects of hydromorphone plus midazolam and hydromorphone plus dexmedetomidine as sedatives following oral and maxillofacial surgery.

## 2. Materials and Methods

### 2.1. Study Design

We had read the Helsinki Declaration and had followed the guidelines in this investigation. This trial was an investigator-initiated randomized clinical trial. The study was prospectively reviewed and approved by the Ethics Committee of the Hospital of Stomatology, Wuhan University. The patients or their relatives have signed the written informed consent forms. Eligible patients were randomly allocated into the midazolam and dexmedetomidine groups. The study has been registered in the Chinese Clinical Trial Registry (ChiCTR1800016017).

### 2.2. Patient Enrollment and Exclusion Criteria

The patients who indwelled nasal tracheal duct in ICU after oral and maxillofacial surgery were enrolled in this study, and they needed sedative treatment. They were aged from 18 years or older and were defined as grade I or II in terms of the American Society of Anesthesiologists. And patients were excluded if they (1) had liver or kidney dysfunction: ALT exceeding 3 times the upper limit of normal as an indicator of liver damage, and serum creatinine and urea nitrogen exceed the upper limit of normal values and are considered indicators of impaired renal function; (2) had acute myocardial infarction or severe heart failure; (3) and had drug dependence or alcoholism or a psychological illness or severe cognitive dysfunction; (4) and were pregnant and lactating; or (5) were allergic to midazolam and dexmedetomidine.

### 2.3. Method of Sedation

All patients recovered spontaneous breathing after surgery and indwelled postoperative intubation into ICU. After admission to ICU, patients were monitored by electrocardiogram, finger oxygen saturation (SpO_2_), mean arterial blood pressure (MAP), heart rate (HR), and respiratory rate (RR), given 2 L/min of oxygen via oxygen insufflation and the holding of the tracheal humidification routinely. Patients in group midazolam (Group M) and group dexmedetomidine (Group D) were both analgesic by hydromorphone and were continuously perfused hydromorphone (4-8 *μ*g/kg/h). Patients in Group M were sedated by midazolam and were continuously perfused midazolam (0.04-0.2 mg/kg/h). Patients in Group D received 1.0 *μ*g/kg of dexmedetomidine over a 10 min period and then continuously perfused dexmedetomidine (0.2-0.7 *μ*g/kg/h).

A Ramsay score should be 2-4 to achieve a satisfactory sedation level, and BPS scores should be ≤4 to achieve a satisfactory analgesic level in our study. According to our local sedation procedure, the nurses continuously monitored the sedation depth and adjusted the dosages of sedative and analgesic drugs to maintain the sedation target level. If the Ramsay score > 4, the nurses increased the infusion rate of dexmedetomidine or midazolam in each group until dexmedetomidine up to a maximum of 1.0 *μ*g/kg/h or midazolam up to a maximum of 0.20 mg/kg/h and recorded the specific Ramsay score. The nurses decreased the infusion rate of dexmedetomidine or midazolam if the Ramsay score < 2. Similarly, they adjusted the infusion rate of hydromorphone based on the BPS score. Ramsay scores and BPS score were recorded before sedation (T0), 30 minutes (T1), 1 hour (T2), 2 hours (T3), 6 hours (T4), and 12 hours (T5) after dexmedetomidine or midazolam initiation in intensive care unit and 10 minutes after extubation (T6) by the nursing staff for statistical analysis. If respiratory depression occurs, immediately assist breathing manually with a simple breathing apparatus connected to oxygen and adjust the sedative and analgesic doses until respiratory depression disappears. According to the needs of patients, flurbiprofen injection (2 mg/kg) was allowed for pain relief in both groups when extubation was decided.

A patient was considered ready for extubation if he recovered pharyngeal airway-protective reflexes and awaked and if a fraction of inspiration O_2_ (FiO_2_) was less than 0.4 and blood oxygen saturation (SpO_2_) exceeded 96%; in addition, tidal volume greater than 6 mL/kg, spontaneous respiratory rate lower than 25/min, the partial pressure of O_2_ in arterial blood (PaO_2_) should be over 80 mmHg, partial pressure of carbon dioxide in the blood (PaCO_2_) below 50 mmHg, with steady circulatory function, and no edema, bleeding, and hemorrhagic secretions observed in the upper respiratory tract.

Clinical indexes included (1) the Ramsay score, the behavioral pain scale (BPS) score, mean arterial blood pressure (MAP), and finger oxygen saturation (SpO_2_); heart rate (HR) and respiratory rate (RR) were recorded before sedation (T0), 30 min (T1), 1 h (T2), 2 h (T3), 6 h (T4), and 12 h (T5) after dexmedetomidine or midazolam initiation in ICU and 10 min after extubation (T6); (2) time to extubation, defined as the time interval between stopping dexmedetomidine or midazolam and the fulfillment of extubation criteria; (3) time to discharge from ICU; (4) the total dosage of analgesic; and (5) potential adverse drug reactions were noted during ICU, which were bradycardia (HR < 50 bpm), tachycardia (HR > 100 bpm), hypotension (mean arterial pressure (MAP) was less than 20% of the baseline), hypertension (MAP was more than 20% of the baseline), and respiratory depression (respiratory rate ≤ 8 bpm or SpO_2_ ≤ 90% for a duration exceeding 5 min).

### 2.4. Statistical Analysis

Before initiating the study, a power analysis suggested that a sample size of 20 patients in each group should be adequate to detect a 30% reduction in extubation time and agitation score with a beta level of 0.2 (80% power) and an alpha level of 0.05, respectively. Continuous variables with a normal distribution are reported as mean and standard deviation (SD). Categorical variables are expressed as frequency and percentage. Student's *t* test was used in the comparisons of age, weight, retention times of nasal tracheal catheter, Ramsay sedation score, and BPS score. The gender and postoperative side effects in two groups were compared using the chi-squared and Fisher's exact tests. A *p* value less than 0.05 was considered statistically significant.

## 3. Results

### 3.1. Patients

**Figure 1:**
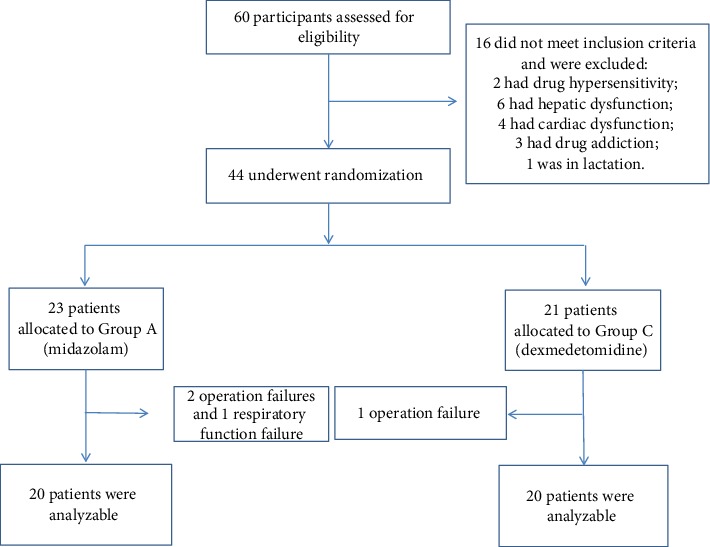
Patient enrollment flow diagram.

All the 60 patients with oral cancer were treated by our surgical team. 16 of these patients did not meet the inclusion criteria: 2 had severe drug hypersensitivity, 6 had hepatic dysfunction, 4 had cardiac dysfunction, 3 had drug addiction, and 1 was in lactation. In total, 44 patients were eligible in this investigation. They were randomly assigned to two groups (M, *n* = 23; and D, *n* = 21) by the use of a computer-generated randomization list. However, there were 3 and 1 patients in midazolam and dexmedetomidine groups who have been ruled out due to operation failure, respectively. 40 out of 60 patients completed the analysis, with 20 subjects in each treatment group, as illustrated in the flow diagram (Figure 1).

### 3.2. Demographic and Surgical Characteristics

The demographic profiles of the patients in all groups were comparable with regard to weight, age, perioperative blood loss, anesthesia time, surgery time, and gender. The two groups were similar in gender, age, body weight, and perioperative blood. There were no significant differences in anesthesia time and operative time between the two groups (Table 1).

### 3.3. Changes of Vital Signs

**Figure 2:**
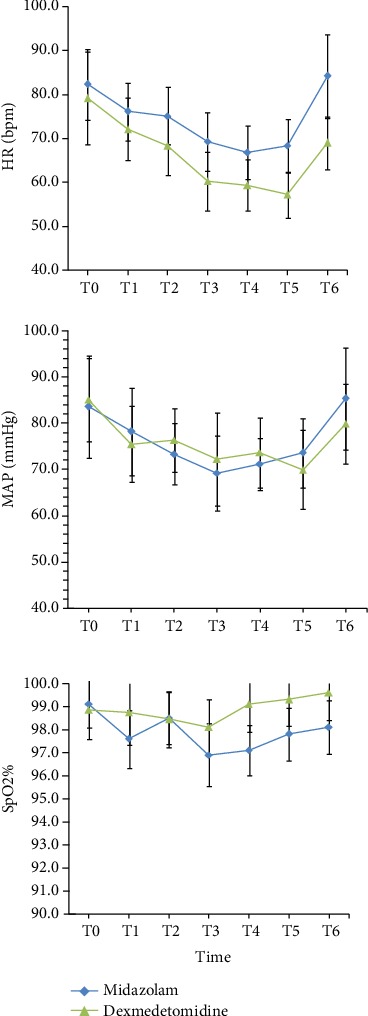
Comparison of heart rate (HR), mean arterial blood pressure (MAP), and the finger oxygen saturation (SpO_2_) between the two groups at each point of time. ^∗^*p* value less than 0.05 was considered statistically significant.

The HR in the midazolam group was stable at T0-T5 while increased after extubation (T6). Dexmedetomidine sedation decreased the HR significantly after its initiation and was significantly less than in the midazolam group at T2, T3, T4, T5, and T6, (*p* = 0.013; Figure 2). In the midazolam group, the MAP was slightly decreased at T1-T5 but increased at the time of extubation (T6) in contrast to dexmedetomidine-sedated patients who were still stable at each time points, but there was not significantly different between the two groups (*p* = 0.087; Figure 2). The SPO_2_ exceeded 95% at each point of time, and it is not significantly different between the two groups at all time points (*p* = 0.108; Figure 2).

### 3.4. Sedative Effect and Analgesic Effect

**Figure 3:**
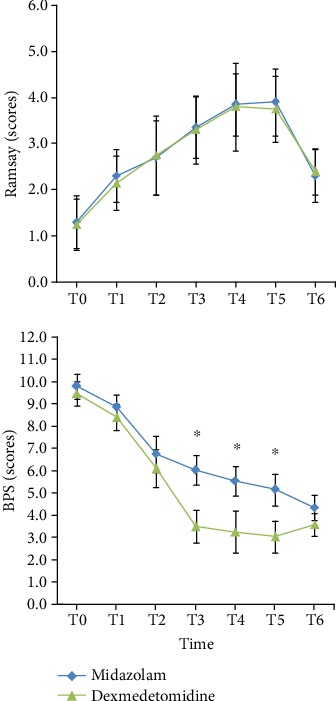
Comparison of the Ramsay score and the behavioral pain scale (BPS) score between the two groups at each point of time. ^∗^*p* value less than 0.05 was considered statistically significant.

Midazolam and dexmedetomidine can meet the needs of ICU patients with sedation. The Ramsay score at all time points can reach 2-4 points after sedation. The Ramsay sedation scores in the midazolam group were higher than those in the dexmedetomidine group at time points of T3-T5; however, there was no significant difference between the two groups as indicated in Figure 3. The results suggested that after admission to ICU, the BPS score in dexmedetomidine group was slightly decreased, then kept at a low level, and was lower than that in the midazolam group at T3-T5. There was no statistically significant difference between the two groups in other time points as indicated in Figure 3.

### 3.5. Side Effects

The dexmedetomidine group required less analgesic than patients in the midazolam group (*p* = 0.001) (Table 2). Patients fulfilled the criteria of extubation earlier in the dexmedetomidine group (*p* = 0.008) (Table 2). There were no statistically significant differences between the two groups in the length of stay in the ICU (*p* = 0.085) (Table 2). No patient was reintubated after extubation. The results showed that the incidence of bradycardia of patients in group D was higher than that in group M (*p* = 0.018) (Table 2). The incidence of hypotension of these two groups was not statistically significantly different (*p* = 0.732) (Table 2). The incidence of respiratory depression of group midazolam was enhanced higher than that of group dexmedetomidine (*p* = 0.037) (Table 2); that is, midazolam may be more likely to cause respiratory depression during sedation compared to dexmedetomidine. The incidence of delirium of group dexmedetomidine was significantly lower than that of group midazolam (*p* = 0.003) (Table 2), i.e., midazolam could increase the possibility of delirium of patients in ICU.

## 4. Discussion

The oral and maxillofacial surgery usually causes the large-scale tissue defects on the mouse, mouse floor and oropharynx. The patients are prone to have respiratory obstruction or even suffocation, endangering the patient's life. Therefore, indwelling endotracheal tube is often required after the surgeries of oral malignant tumor. However, the tracheal tube is also a source of stimulation, and retention of tracheal catheter can cause patients' cough, agitation, increased blood pressure and heart rate, and other adverse reactions [7]. These nociceptive stimuli not only adversely affect the body's physiological function but also significantly delay the patient's recovery process and even can produce more serious surgical complications. Therefore, taking an effective sedative analgesic drug to block these stress responses and physiological changes is essential. In the ICU of our department, we administrated dexmedetomidine and midazolam to patients after oral and maxillofacial surgery for sedation.

The America adult ICU guideline (2013 version) proposes that the primary factor for patients' agitation is pain and discomfort, which is treated by opioids as the most basic treatment [8]. Recent researches indicate that providing “analgesic priority” treatment program can effectively improve the patient's comfort for the ICU patients [9–12]. Compared with the traditional sedative treatment, this treatment is beneficial for reducing the patient's stay in the ICU [13]. Therefore, in our study, patients of the two groups were both treated with opioid analgesics and continuous perfused analgesia with hydromorphone, and then, sedation is achieved with midazolam and dexmedetomidine. Finally, the efficacy and safety of various drugs are compared and observed. Hydromorphone is an a*μ*-opioid receptor agonist, which can well depress the adverse reaction of the cardiovascular system caused by stress reaction. And it can maintain the hemodynamic stability and have mild respiratory depression [6, 14].

Although the sedation mechanisms of midazolam and dexmedetomidine are different, our study indicates that they both provide a good sedative effect on patients who have oral and maxillofacial surgery. The Ramsay scores of patients reach 2-4, and there was no statistically significant difference in sedation depth for the two drugs. In respect of the analgesic effect, studies have shown that dexmedetomidine can be safely used more than 72 h, significantly reducing the analgesic demand with 50%~70% compared with propofol [15, 16]. Our results also show that the dexmedetomidine group required less analgesic than the midazolam group, so dexmedetomidine may have a certain degree of analgesic effect. The midazolam group was required to pump a larger dosage of hydromorphone. It indicates that dexmedetomidine may have a certain degree of analgesic effect that reduces the side effects of opioid analgesics, such as respiratory depression and other complications [17]. The incidence of respiratory depression was significantly higher in the midazolam group than in the dexmedetomidine group in our study.

We regard systolic pressure < 90 mmHg (1 mmHg = 0.133 kPa), diastolic pressure < 60 mm Hg, heart rate < 60 beat/min, or the changes in blood pressure and heart rate were greater than 20% compared with baseline as adverse events of hypotension and bradycardia. Our results show that the incidence of bradycardia of patients in group dexmedetomidine was higher than that in group midazolam. The hypotension probability is almost the same, and the difference is not statistically significant.

The time to extubation of patients after discontinuation of the drug was shorter in the dexmedetomidine group than that in the midazolam group, and the difference was statistically significant. There were no statistically significant differences between the two groups in the length of stay in the ICU. The incidence of delirium of group dexmedetomidine was significantly lower than that of group midazolam. Dexmedetomidine can reduce the dose of GABA drugs and may be the cause of the decrease in delirium [18].

## 5. Conclusion

In summary, based on the use of opioids for fully analgesia, midazolam and dexmedetomidine can provide good sedation for patients with retention of tracheal intubation after oral and maxillofacial surgery. Dexmedetomidine may have a certain degree of analgesic effect and thus can reduce the opioid dosage. In respect of adverse reactions, the incidence of bradycardia of patients in group dexmedetomidine was higher than that in group midazolam. In respect of respiratory depression, dexmedetomidine reduces the incidence of respiratory depression of patients. And compared with midazolam, dexmedetomidine can significantly reduce the incidence of delirium of patients in ICU. However, whether assessment of patient sedation is appropriate depends not only on patients' comfort but also on the resource consumption and mortality. Besides, it is needed to assess its long-term cognitive and psychological sequel in addition to the recovery of the primary disease for a better understanding of the prognosis of the patient. Clinically, we should avoid the adverse effects caused by the use of drugs as well as reduce the pain of patients, so as to ensure that patients spend the convalescence safely. These are the points without full discussion in this paper.

## Figures and Tables

**Table 1:** Demographic and surgical date (*n* = 20).

	Group M (*n* = 20)	Group D (*n* = 20)
Gender (M/F)	15/5	14/6
Age (years)	60.50 ± 8.19	60.05 ± 10.10
Weight (kg)	63.90 ± 8.31	68.40 ± 9.07
Blood loss (mL)	476.75 ± 68.81	479.00 ± 67.41
Duration of anesthesia (h)	7.33 ± 0.67	7.48 ± 0.99
Duration of surgery (h)	6.56 ± 0.94	6.61 ± 0.99

Baseline characteristics of the patients receiving dexmedetomidine or miadazolam sedation. Values are mean ± SD or numbers. Differences were not statistically significant.

**Table 2:** The dosage of analgesic, time to extubation and ICU stay, and the incidence of side effects.

	Group M (*n* = 20)	Group D (*n* = 20)
The dosage of analgesic (mg)	5.69 ± 0.77	3.87 ± 1.13^∗^
Time to extubation (min)	17.68 ± 3.41	11.36 ± 1.00^∗^
Length of ICU stay (h)	14.68 ± 2.56	13.36 ± 2.03
Respiratory depression	7 (35%)	1 (5%)^∗^
Bradycardia	2 (10%)	8 (35%)^∗^
Hypotension	3 (15%)	4 (20%)
Delirium	9 (45%)	1 (5%)^∗^

Values are mean ± SD or numbers, ^∗^*p* value less than 0.05 was considered statistically significant.

## Data Availability

The data used to support the findings of this study are available from the corresponding author upon request.
